# Impaired impulse inhibition of emotional stimuli in patients with borderline personality disorder

**DOI:** 10.1038/s41598-021-96166-1

**Published:** 2021-08-17

**Authors:** Huihui Yang, Qian Liu, Wanrong Peng, Zhaoxia Liu, Jun Chu, Kaili Zheng, Wanyi Cao, Jinyao Yi

**Affiliations:** 1 Medical Psychological Center, The Second Xiangya Hospital, Central South University, Changsha, Hunan 410011 People’s Republic of China; 2 Medical Psychological Institute, Central South University, Changsha, Hunan 410011 People’s Republic of China; 3 National Clinical Research Center for Mental Disorders, Changsha, Hunan 410011 People’s Republic of China

**Keywords:** Neuroscience, Psychology

## Abstract

This study was aimed to investigate whether BPD patients showed impaired impulse inhibition of emotional and non-emotional stimuli and to explore relevant neuroelectrophysiological mechanisms. A total of 32 BPD patients and 32 matched healthy controls were recruited. Self-reported scales were used to measure psychiatric symptoms. The event-related potentials (ERPs) were recorded when subjects were performing neutral and emotional Stop Signal Task (SST). Group differences in self-reported scores, behavioral variables and ERPs were compared. The BPD group scored significantly higher on impulsivity, severity of BPD symptoms, levels of depression and anxiety than the control group. In neutral SST, no significant group differences were detected in the amplitude and latency of ERPs components induced. In emotional SST, the P2 amplitude of negative emotion was significantly larger than that of neutral emotion in Go trials. In Stop trials, the P2 amplitude of BPD group was significantly smaller than that of control group, and the N2 amplitude of BPD group was significantly greater than that of control group. BPD patients showed impaired inhibition of emotional stimuli rather than non-emotional stimuli. The deficits of emotional impulse control mainly exhibit at the early attention, stimulus evaluation and conflict detection stages.

## Introduction

Borderline Personality Disorder (BPD) is a complex psychiatric disorder characterized by persistent serious impairment of cognitive, behavioral, interpersonal, and emotional functions^1–3^. As one of the core features of BPD, impulsivity is considered to be a clinical, diagnostic, and pathophysiological marker of BPD. Impulsivity manifests through careless behaviors without considering long-term consequences, which may lead to suicide, self-mutilation, substance abuse, overeating and other adverse behavioral consequences^1^. In a seven-year follow-up study, the impulsiveness of BPD was stable and highly predictive of the psychopathological features of borderline symptoms^4^.

A line of research has explored whether the impulsive behavior problems of BPD patients are caused by their defects in impulsive inhibition. In Stroop tasks, researchers reported no significant group difference between BPD patients and healthy controls in color-word^5^ and emotional word Stroop tasks^6^, but BPD patients did show significant Stroop effect in the facial emotional Stroop task compared with control subjects^7^, implying that emotional faces might impede the interference inhibition of BPD patients. In delayed discount task and probabilistic discount task, researchers reported that BPD patients were more likely to accept timely smaller rewards rather than to wait for larger rewards, indicating that BPD patients are impulsive in decision-making^8,9^. In Stop Signal Task (SST), researchers found that there were significant correlations between response inhibition and BPD symptoms even after controlling a number of variables^10^. In Immediate Memory Task (IMT), researchers reported that BPD group showed more motor impulsivity and higher scores of Barratt Impulsiveness Scale than control group^11^. The results of SST and IMT suggested that BPD patients showed deficits in action inhibition. In the Go/Nogo task and continuous performance test, researchers reported that BPD patients made more commission errors than healthy controls^12–14^, which suggested that BPD patients had difficulties in suppressing dominant responses^15,16^. Therefore, previous researches suggested that the impulsive behaviors of BPD patients are caused by their impaired impulsive inhibition.

BPD patients are emotionally sensitive, manifesting through lower thresholds for identifying emotional stimuli, greater emotional responses, greater difficulties in regulating emotion, and longer emotional response time^13,17,18^. High emotional sensitivity of BPD patients may induce more frequent, intense and lasting emotional experiences and may contribute to other clinical features (e.g., impulsivity, self-harm, and aggressive behaviors) in BPD patients^19^. Empirical studies have demonstrated that compared with control subjects, BPD patients reported more negative emotions and more impulsive symptoms than healthy controls^11,20,21^. One study found that BPD patients showed higher accuracy in recognizing facial expressions than the control group^22^, suggesting that BPD patients are more sensitive to emotional stimuli. Another study found that BPD patients tended to recognize neutral facial expressions as negative expressions when required to discriminate negative and neutral facial expressions^23^. BPD patients also have difficulties in identifying angry faces and tend to recognize other facial expressions as angry^24^, which might explain their misunderstanding of others’ emotions in social situation. Also, one study showed that emotional intensity and the lack of affect control were positively correlated with BPD features after controlling depression^25^, indicating that BPD patients experience stronger emotion and have more difficulties in inhibiting reaction to emotional stimuli. Inhibition is the main mechanism of emotion regulation, which involves, for example, shifting attention away from emotional disturbances and actively suppressing emotional responses. Therefore, BPD patients might have deficits in response inhibition of emotional stimuli. In directed forgetting task and emotional negative-prime task, Domes et al., (2006) found that BPD patients showed significantly reduced inhibition of negative stimuli than healthy controls^6^, suggesting the inability of response inhibition of negative emotion in BPD patients.

Researchers have been exploring the neural mechanism of impaired inhibition in BPD. Previous neuroimaging studies have revealed that, compared with the healthy controls, BPD patients showed reduced activation in the left frontal cortex^26^ and prefrontal cortex (PFC)^27^ in Nogo trials of Go/Nogo task. As the prefrontal cortex was related to successful inhibition, the results revealed that the deficits in inhibitory control of BPD patients were accompanied by specific brain function disturbances^28^. The frontal and parietal disruption in BPD was also associated with response inhibition and attentional saliency, indicating that neurological dysfunction in BPD is related to attention for salient or infrequent stimuli^29^. In Event Related Potentials (ERPs) studies, researchers found that the amplitudes of the N1/P2 components of BPD patients increased significantly with the increase of the stimuli intensity, accompanied by decreased latency, which meant that the inhibitory control of BPD patients was weakened^30^. Induced in Nogo or Stop trials, N2, a component reflecting conflict monitoring^31–34^, was consistently found not different in N2 amplitude between BPD patients and controls in Go/Nogo task^35,36^ and stop signal task^37^ in previous studies. As for P3 component, BPD patients showed decreased Nogo-P3 amplitude compared with control subjects, and a negative correlation existed between cognitive impulsiveness and Nogo-P3 amplitude, indicating that the higher cognitive impulsivity BPD patients experienced, the smaller amplitude of Nogo-P3 was^35^. However, in emotional Go/Nogo tasks, the Nogo-P3 amplitude was significantly higher in BPD group than in control group^38^, and high-impulsive individuals had greater amplitudes of Nogo-P3 for emotional stimuli than for neutral stimuli^36^, indicating that impulsivity might affect the response inhibition of emotional stimuli. As a component reflecting error detection and impulsivity^39^, the amplitude of error related negativity (ERN) was reduced in BPD patients, which suggested that BPD patients had impaired error monitoring^40,41^. In other words, BPD patients could not adjust their behavior from the mistakes and would maintain their impulsive response style^40,41^.

Summing up the results of previous studies, we still cannot clarify whether BPD patients manifest impaired impulse inhibition only when facing negative emotional information or facing both emotional information and non-emotional information. Meanwhile, most of the existing studies on BPD inhibitory control ability adopted the Go/Nogo task but not stop signal task (SST). Although both Go/Nogo task and SST measure response inhibition, there are still differences between the two tasks. Researchers have discriminated the inhibition measured by Go/Nogo task and SST conceptually. The stimulus and required response in Go/Nogo task are always paired, and no further executive control is needed, which implies automatic bottom-up inhibition. However, the stimulus and required response in SST are inconsistently paired and rely on additional executive control, which reflects controlled top-down inhibition^42–44^. In summary, considering the different inhibition measured by Go/Nogo task and SST, it is necessary to adopt SST to investigate the inhibitory control in BPD patients, which will be helpful to understand the mechanism of impaired impulse inhibition in BPD patients.

Therefore, by adopting the stop signal task, this study was aimed to investigate whether BPD patients would show impaired impulse inhibition of neutral and emotional stimuli. Two types of SST were used in this study: the neutral SST, whose stimuli did not contain any emotional features, and the emotional SST, whose stimuli were negative and neutral emotion face pictures. Event-related potentials were also acquired to explore the neuroelectrophysiological mechanism of impaired impulse inhibition in BPD patients. We predict that compared with control group, BPD patients would show impaired inhibitory control to emotional stimuli, their inhibitory performances of negative emotional stimuli would be better than those of controls, and their ERP components induced in Stop trials in emotional SST would be different from those of controls.

## Results

### Self-reported results

As Table 1 shows, the BIS total scores of BPD group were significantly higher than those of control group. BPD group had significantly greater scores on attentional impulsivity and motor impulsivity than control group, but there was no significant group difference in non-planning impulsivity. There were significant group differences in scores of BSL-23, CES-D, and STAI (*p* < 0.01), and BPD group had significantly higher scores of the above scales than control group. The Cohen's *d* value ranged from 0.28 to 2.60.

**Table 1:** Differences on scores of self-reported scales between BPD group and control group(M ± SD).

	BPD group (n = 32)	Control group (n = 32)	*t*	*p*	|Cohen’s *d*|
BIS total score	72.06 ± 11.97	65.16 ± 8.07	3.10	0.003	0.68
BIS-ATT	20.75 ± 3.76	16.91 ± 2.29	4.94	0.000	1.23
BIS-MOT	23.72 ± 4.91	21.03 ± 3.79	2.45	0.017	0.61
BIS-NP	28.59 ± 5.61	27.21 ± 4.04	1.13	0.265	0.28
BSL-23	41.47 ± 16.18	8.00 ± 8.42	10.38	0.000	2.60
CES-D	48.91 ± 9.09	33.43 ± 9.24	6.75	0.000	1.69
STAI-S	42.44 ± 9.88	35.94 ± 7.20	3.01	0.004	0.75
STAI-T	51.19 ± 7.83	39.53 ± 7.01	6.27	0.000	1.57

BPD, borderline personality disorder; BIS, Barratt Impulsivity Scale; BIS-ATT, Attention Impulsivity subscale of Barratt Impulsivity Scale; BIS-MOT, Motor Impulsivity Subscale of Barratt Impulsivity Scale; BIS-NP, Non-Planning Impulsivity Subscale of Barratt Impulsivity Scale; BSL-23, Borderline Symptom List; CES-D, Center for Epidemiologic Studies Depression Scale; STAI-S, State Anxiety Inventory; STAI-T, Trait Anxiety Inventory; |Cohen’ s *d*|, absolute value of Cohen’ s *d.*

### Behavioral results

In the neutral SST, the independent sample *t*-test results showed that there were no significant group differences in Go-ACC, Go-RT, Stop-ACC, SSD, SSRT (*p* > 0.05; Table 2).

**Table 2:** Differences on performance in neural SST between BPD group and control group (M ± SD).

	BPD group (n = 32)	Control group (n = 32)	*t*	*p*	|Cohen’s *d*|
Go-ACC	0.90 ± 0.12	0.90 ± 0.16	− 0.15	0.89	0.00
Go-RT (ms)	651.11 ± 129.72	678.66 ± 111.70	− 0.91	0.37	0.23
Stop-ACC	0.71 ± 0.18	0.72 ± 0.16	− 0.32	0.75	0.06
SSD (ms)	327.09 ± 90.87	342.66 ± 75.36	− 0.75	0.46	0.19
SSRT (ms)	324.01 ± 76.36	335.99 ± 53.18	− 0.73	0.47	0.18

Go-ACC, the correct rate of Go trials; Go-RT, the response time of Go trials; Stop-ACC, the correct rate of Stop trials; SSD, stop signal delay; SSRT, stop signal reaction time; |Cohen’ s *d*|, absolute value of Cohen’ s *d.*

For the emotional SST, the correct rate and response time are shown in Table 3. Repeated-measures ANOVA showed that the group differences were significant in Go-ACC. The Go-ACC of BPD group was significantly higher than that of control group (F_(1,62)_ = 4.07, *p* = 0.048 , partial η^2^ = 0.06). The main effect of emotion type was significant in the Go-ACC (F_(1,62)_ = 101.73, *p* = 0.000, partial η^2^ = 0.62). The interaction between group and emotion type was significant (F_(1,62)_ = 4.07, *p* = 0.048, partial η^2^ = 0.06). Simple effect test showed that the Go-ACC of BPD group was significantly higher than that of control group under neutral condition, while there was no significant group difference in Go-ACC under negative emotion condition. In both groups, the correct rates of negative emotion faces were significantly lower than those of neutral emotion faces. For the Go-RT, Stop-ACC, SSD, and SSRT, none of the group differences, the main effects and interactions between group and emotion type were significant (*p* > 0.05, partial η^2^ < 0.06).

**Table 3:** Performance of BPD group and control group in emotional SST (M ± SD).

	BPD group(n = 32)		Control group(n = 32)	
	Fear emotion	Neutral emotion	Fear emotion	Neutral emotion
Go-ACC	0.80 ± 0.08	0.89 ± 0.07	0.76 ± 0.14	0.82 ± 0.15
Go-RT (ms)	681.75 ± 78.82	679.78 ± 77.39	690.65 ± 65.37	687.37 ± 66.15
Stop-ACC	0.67 ± 0.15	0.68 ± 0.16	0.67 ± 0.15	0.68 ± 0.13
SSD (ms)	337.66 ± 73.74	334.53 ± 75.13	341.32 ± 55.65	342.21 ± 52.74
SSRT (ms)	344.10 ± 48.96	345.25 ± 40.69	339.33 ± 40.60	345.15 ± 40.57

Go-ACC, the correct rate of Go trials; Go-RT, the response time of Go trials; Stop-ACC, the correct rate of Stop trials; SSD, stop signal delay; SSRT, stop signal reaction time.

### ERPs results

#### ERPs results of neutral SST

**Figure 1:**
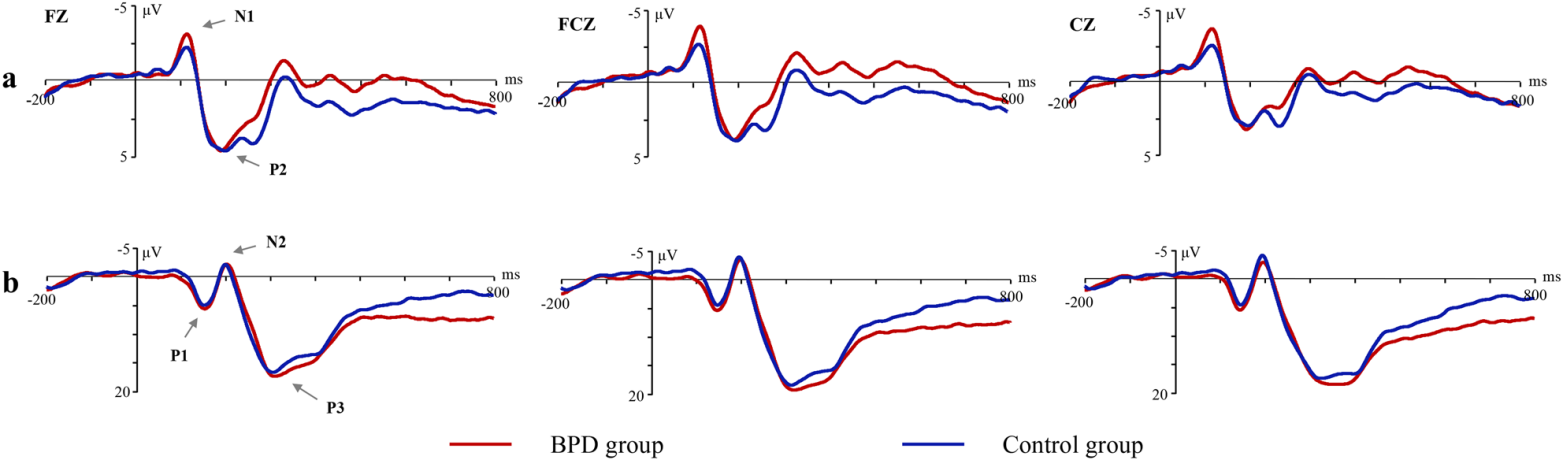
ERPs waveform in neutral stop signal task [(**a**) Go trials; (**b**) Stop trials].

As Fig. 1 shows, N1 and P2 were induced in the Go trials of the neutral SST, while three components (P1, N2 and P3) were induced in the Stop trials of the neutral SST. The group differences in all components’ amplitude and latency were insignificant (*p* > 0.05, partial η^2^ < 0.06). All interactions were insignificant.

#### ERPs results of emotional SST

**Figure 2:**
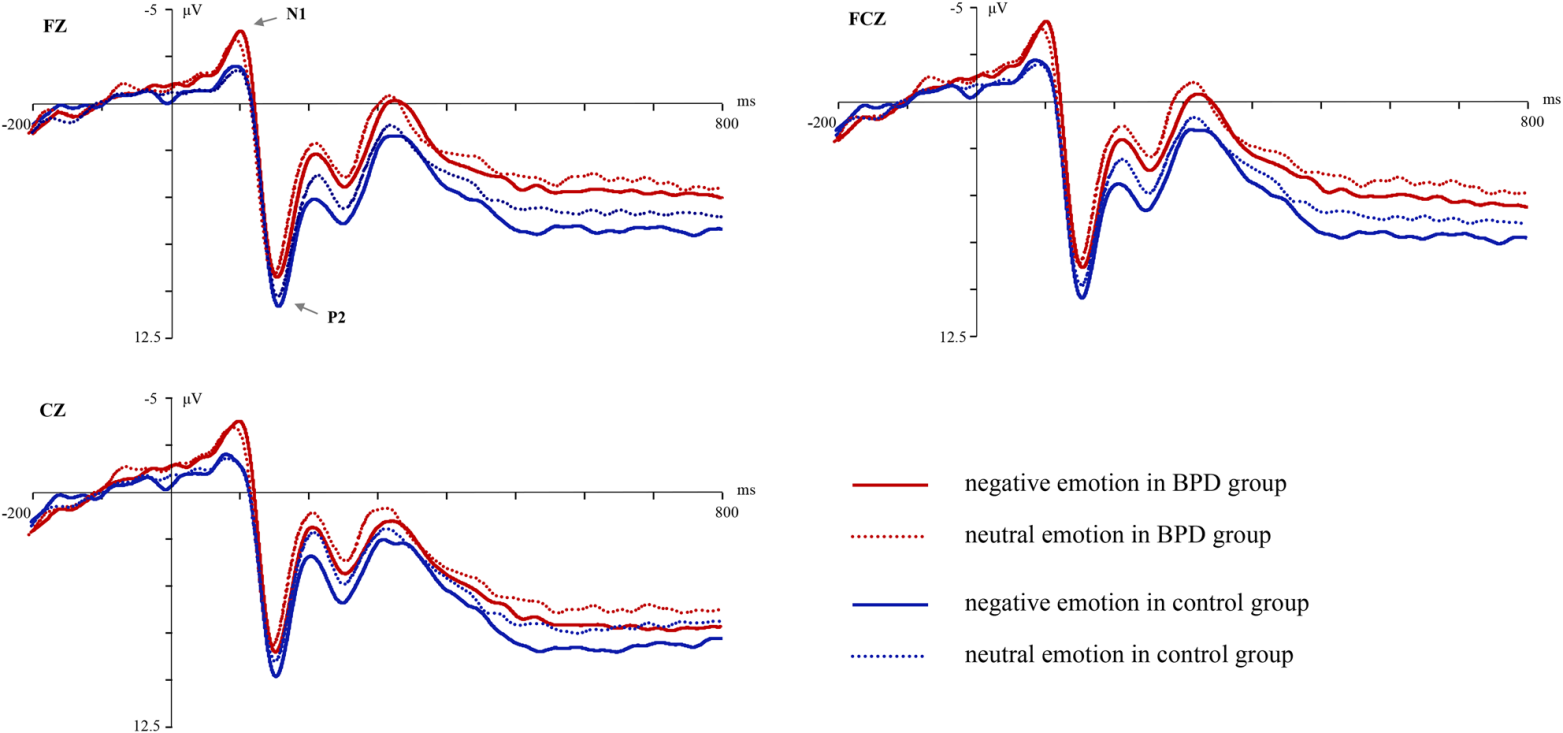
ERPs waveform of Go trials in emotional stop signal task.

For the Go trials in the emotional SST, N1 and P2 were induced. The main effect of emotion type was significant in P2 amplitude (F_(1,60)_ = 9.43, *p* = 0.03, partial η^2^ = 0.13); the P2 amplitude of negative emotion was significantly larger than that of neutral emotion. But the group difference and the group × emotion type interaction were not significant in P2 amplitude. For the P2 latency, none of the effects were significant. For the N1 component, the group differences in the amplitude and latency were not significant, neither the main effect of emotion type nor any interactions (see Fig. 2).

**Figure 3:**
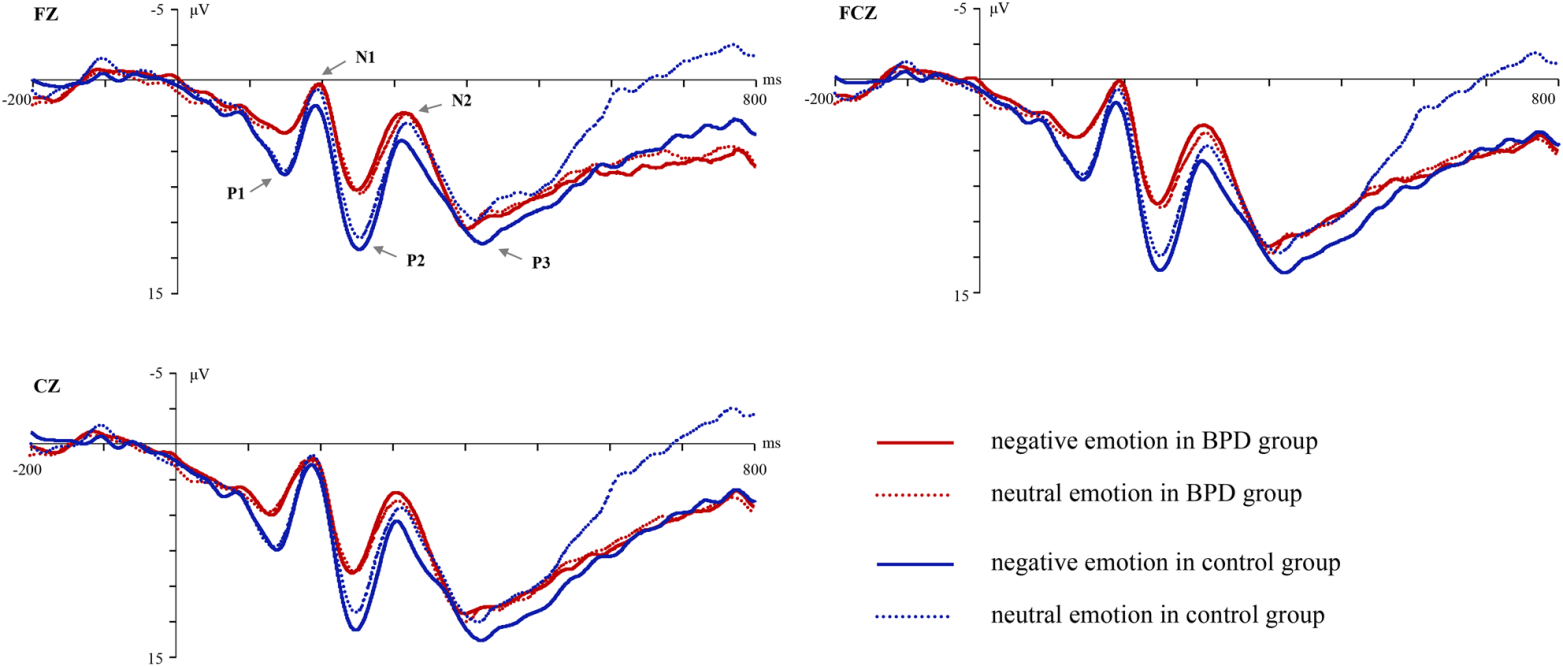
ERPs waveform of Stop trials in emotional stop signal task.

For the Stop trials in the emotional SST, five components (P1, N1, P2, N2 and P3) were induced. The group difference in P2 amplitude was significant (F_(1,60)_ = 4.65, *p* = 0.035, partial η^2^ = 0.07). The P2 amplitude of BPD group was smaller than that of the control group, but the main effect of the emotional type was not significant. The group difference in N2 amplitude was significant (F_(1,60)_ = 4.02, *p* = 0.049, partial η^2^ = 0.06). The N2 amplitude of BPD group was significantly more negative than that of control group. None of the effects of P2 and N2 latencies were significant. No effects of P1, N1 and P3 amplitudes and latencies were significant (see Fig. 3).

## Discussion

High impulsivity and emotional sensitivity are two closely related symptoms of BPD. In this study, the impulse inhibition of neutral stimuli and negative emotional stimuli in BPD patients were both investigated with neutral and emotional SST. The ERPs components were examined to explore the time course of the inhibitory control process and the neuroelectrophysiological mechanism of the impaired impulse inhibition in BPD. This study found that BPD patients showed inhibition control deficits for emotional stimuli rather than for non-emotional stimuli, which emerged in the early attention distribution, stimuli evaluation and conflict monitoring stages of negative information processing. This study enriched existing research findings and provided extra evidence about impaired inhibition control of emotional stimuli in BPD, which further suggests for intervention of the impulsive behaviors in BPD.

In this study, the BPD group scored significantly higher on the attention impulsiveness and motor impulsiveness than the control group, while there was no significant group difference in non-planning impulsiveness. Attention impulsiveness refers to poor cognitive control and inattention, motor impulsiveness refers to behaviors without thinking, and non-planning impulsiveness refers to thoughtless decision-making^45^. Our results suggested that BPD patients showed weaker cognitive control and more impulsive behaviors, but their decision-making was intact. The BPD group had significantly higher scores on BSL-23 than the control group, which validated the more serious symptomatology in BPD. This study found that the BPD group had both significantly greater scores of CES-D and STAI than the control group, which confirmed that BPD patients had higher levels of depression and anxiety^46,47^.

In the emotional SST, there was significant group and emotion type interaction on Go-ACC. In both groups, the Go-ACC of negative faces was less than that of neutral faces, indicating that negative emotions might interfere the task performance. Meanwhile, the effect size in BPD patients was greater than that in the control subjects, supporting that BPD patients were more sensitive to the negative emotional stimuli than control subjects. Previous studies found that humans are biased toward negative emotions^48^, which means that people are more likely to notice negative information than neutral information. Researchers have found that task performance for neutral emotional stimuli is better than that for negative emotional stimuli^49,50^, which is consistent with our results. In this study, the correct rates of negative faces of all subjects were less than those of neutral faces, which suggested that more attention resources were allocated to the negative faces because of the negative bias, resulting in decreases in attention resources allocated to the gender judgment process required by the task.

As an indicator of inhibition ability, smaller SSRT indicates stronger response inhibition ability^51^. In this study, there was no significant group difference in SSRT in the neutral SST, which was consistent with results of previous studies^5,8,52^. No significant group differences in the amplitude and latency of all ERPs components were found, which were inconsistent with previous results^35,37^. Using Go/Nogo task, Ruchsow et al. (2008)^35^ found that the amplitude of Nogo-P3 was lower in BPD patients than that in healthy controls, while there was no group difference in Nogo-N2 component. Using a letter-based SST paradigm to investigate the effect of impulsive trait on response inhibition, Shen et al. (2014)^37^ found that the P3 amplitudes of highly impulsive individuals were smaller than those of low impulsive individuals. It should be noted that in Shen’s SST study, all participants were males^37^, while in the present study, a large proportion of the participants were females. The gender difference in inhibitory control might be a potentially confounding factor for the inconsistent results. Our study found no significant group differences in N2 and P3 components of Stop trials. Therefore, in combination with the behavioral results of the neutral SST, our results suggested that the impulse inhibition of neutral stimuli in BPD patients might not be impaired.

In the Go trials of emotional SST, this study found that the P2 amplitude of negative faces was larger than that of neutral faces. Previous studies have also found that P2 amplitude induced by negative pictures was significantly larger than that induced by positive pictures^49,53^. As a component related to attention, the increased P2 amplitude suggested that BPD patients might assign more attention resources to negative faces and show greater preference for negative stimuli^48^.

In the Stop trials of emotional SST, the P2 amplitude of BPD group was significantly smaller than that of the control group. The Stop-P2 component reflected the early attention stage and task-related stimulus evaluation process, which was essential for optimizing the current task performance^48,54^. As P2 is sensitive to task requirement, less frequent stimuli can trigger larger P2 amplitude^55^. During a series of stimuli presented, subjects develop dominant responses to frequent stimuli, and they need to evaluate the stimulus to make the correct response when dealing with less frequent stimuli. In this study, the stop signal was a non-dominant response stimulus, which required subjects to assign more attention to the stop signal and ignore other stimuli, so as to process and evaluate the target stimuli and respond accurately. The decrease in the P2 amplitude of the BPD group compared with the control group suggested that the BPD patients had impaired ability to make the early attention distribution of non-expected stimuli and assess the task-related stimuli, which then might affect the subsequent impulse inhibition and lead to failure of inhibition. Therefore, the defects of emotional information processing in early attention and assessment stage might be the basis of inhibition impairment in BPD.

In the Stop trials of emotional SST, the BPD group also had more negative N2 amplitude than control group. Although some studies did not find significant differences in N2 amplitude between BPD patients and controls in Go/Nogo task^35,36^ or stop signal task^37^, several studies suggested that the N2 component in Nogo or Stop trials was related to conflict monitoring^31–34^. For example, N2 in the Nogo task reflects the competition conflict caused by response and inhibition^34^. This study found that the N2 amplitude of the BPD group was significantly greater than that of the control group in Stop trials, supporting that BPD group might experience greater conflict between Go response and Stop response than the control group. The N2 latency in Stop trials showed no significant group difference, suggesting that the impaired conflict monitoring ability of BPD patients was mainly reflected by the difficulty in conflict assessment, rather than the speed of stop signal perception.

In this study, no significant group difference was found in P3 component, while some previous studies found that the Stop-P3 amplitude under successful inhibition condition was greater than that under failure condition^56–58^, suggesting that P3 component in Stop trials was associated with successful inhibition. This study did not find significant group difference in the amplitude of Stop-P3, probably because the probability of successful inhibition in Stop trials was so high as to cover the effect of P3. Future studies need to focus on the differences in EEG activity between successful inhibition and failed inhibition and explore the brain mechanism of impulse inhibition in BPD patients.

In this study, the impaired inhibition control ability of BPD patients was demonstrated in the emotional SST. Specifically, BPD patients showed deficits in focusing on unexpected stop signal at the early attention distribution stage, and had difficulty in conflict assessment when faced with stop signals. However, we did not find impaired inhibition control of BPD patients in the neutral SST, suggesting that the deficits in impulse inhibition of BPD patients were mainly reflected in the process of inhibition control of emotional stimuli.

There were still several limitations in this study. Firstly, the neutral SST used in this study was relatively simple, which might lead to high correct rate and the “ceiling effect”. Future research should improve the neutral SST by using more types of stimuli or more complex reaction rules to further validate the results of this study. Secondly, this study only adopted fearful faces to examine the impulse inhibition of negative emotional stimuli in BPD. Future research can use more types of negative stimuli (such as threatening pictures, sad face pictures, etc.) to examine the impulse inhibition of negative stimuli more comprehensively and deeply. Thirdly, this study did not include a clinical control group, so it’s not clear whether the findings are specific to BPD or common in clinical patients with high impulsivity or deficits in inhibitory control, such as patients with bipolar disorder or attention deficit hyperactivity disorder^59,60^. Future studies should recruit other types of clinical patients to be control groups. Finally, the participants in this study were not asked to assess the valence of emotional faces used in emotional SST. As BPD patients might tend to perceive neutral emotions as negative emotions^23^, further studies should select more appropriate neutral materials according to the ratings of BPD patients.

## Conclusion

BPD patients showed higher levels of impulsivity, depression and anxiety than control group. Both BPD patients and control subjects showed more attention to negative emotion stimuli and manifested negative bias. In the time course of information processing, BPD patients showed reduced ability to suppress reaction to emotional stimuli rather than to non-emotional stimuli. The impaired inhibition control in BPD patients was manifested at the early attention and stimuli evaluation stages of emotion processing, and greater conflicts between response and inhibition were experienced by BPD patients during the conflict monitoring phase.

## Methods and materials

### Subjects

The BPD patients were recruited from the medication free outpatients in the psychological clinics and psychiatric clinics of two hospitals. Diagnoses were made by two trained clinical psychiatrists using the Structured Clinical Interview for axis II disorders (SCID-II) of the Diagnostic and Statistical Manual of Mental Disorders, Fourth Edition (DSM-IV). All BPD patients were also interviewed with SCID-I to exclude current and past Axis I disorders, such as major depression disorder, bipolar disorder, schizophrenia and attention deficit hyperactivity disorder.

The control group subjects, matched with age, gender and intelligence level with the BPD group, were recruited from local community. The controls were interviewed by two clinical psychiatrists with SCID I and II to exclude BPD and any other axis I/II disorders.

All participants also completed the abbreviated version of the Wechsler Adult Intelligence Scale-Chinese Revision (WAIS-RC) to assess the general intelligence and exclude mental retardation^61^. Participants with history of head injury or nerve system diseases, or current medical problems were also excluded.

This study was conducted in accord with the declaration of Helsinki and approved by the Ethics Committee of Central South University of Second Xiangya Hospital. All participates agreed to participate in this study. Informed consent was obtained from participants or one of their parents.

A total of 32 BPD patients (19.19 ± 1.18 years old; 26 females vs. 6 males; IQ = 107.36 ± 10.12) and 32 control subjects (19.44 ± 1.78 years old; 21 females vs. 11 males; IQ = 110.36 ± 11.02) completed the procedures in this study.

### Instruments

#### Barratt Impulsiveness Scale, 11th version (BIS-11)

The BIS-11 contains 30 items with a 1–4 four-level rating scale (1 = never, 2 = occasionally, 3 = often, 4 = always). The total score is in the range of 30–120. The BIS-11 consists of three subscales, namely attention impulsivity (8 items), which means poor cognitive control and lack of concentration; motor impulsivity (11 items), which means showing no thinking on actions; non-planning impulsivity (11 items), which means bare consideration in decision making^45^. The higher score of one subscale indicates that the corresponding impulsivity type is more obvious. The Chinese version of BIS-11 has shown good reliability and validity^62^.

#### Borderline Symptom List (BSL-23)

The BSL-23 contains 23 items, using Likert 5-point scoring method (0 = no feelings, 4 = very strong). The total score is in the range of 0–92. Higher score suggests greater severity of borderline symptoms. Our previous study supported that the Chinese version of BSL-23 has good reliability and validity^63^.

#### Center for Epidemiologic Studies Depression Scale (CES-D)

The CES-D has 20 entries with a Likert 4-point scale (1 = no or almost no, 2 = slightly, 3 = often, 4 = almost always). The total score ranges from 20 to 80. Higher score indicates greater severity of depressive symptoms. The Chinese revised version of CES-D has good psychometric properties^64^.

#### State-Trait Anxiety Inventory (STAI)

The STAI has a total of 40 items with a Likert 4-point scale (1 = no, 2 = some, 3 = moderate, 4 = very obvious), which contains two subscales evaluating state anxiety and trait anxiety. Each subscale has 20 items, and the total score of each subscale ranges from 20 to 80. Higher score indicates greater severity of the corresponding anxiety type. The Chinese version of STAI has been evidenced with satisfied reliability and validity^65^.

### Experiment tasks

#### Neutral SST

**Figure 4:**
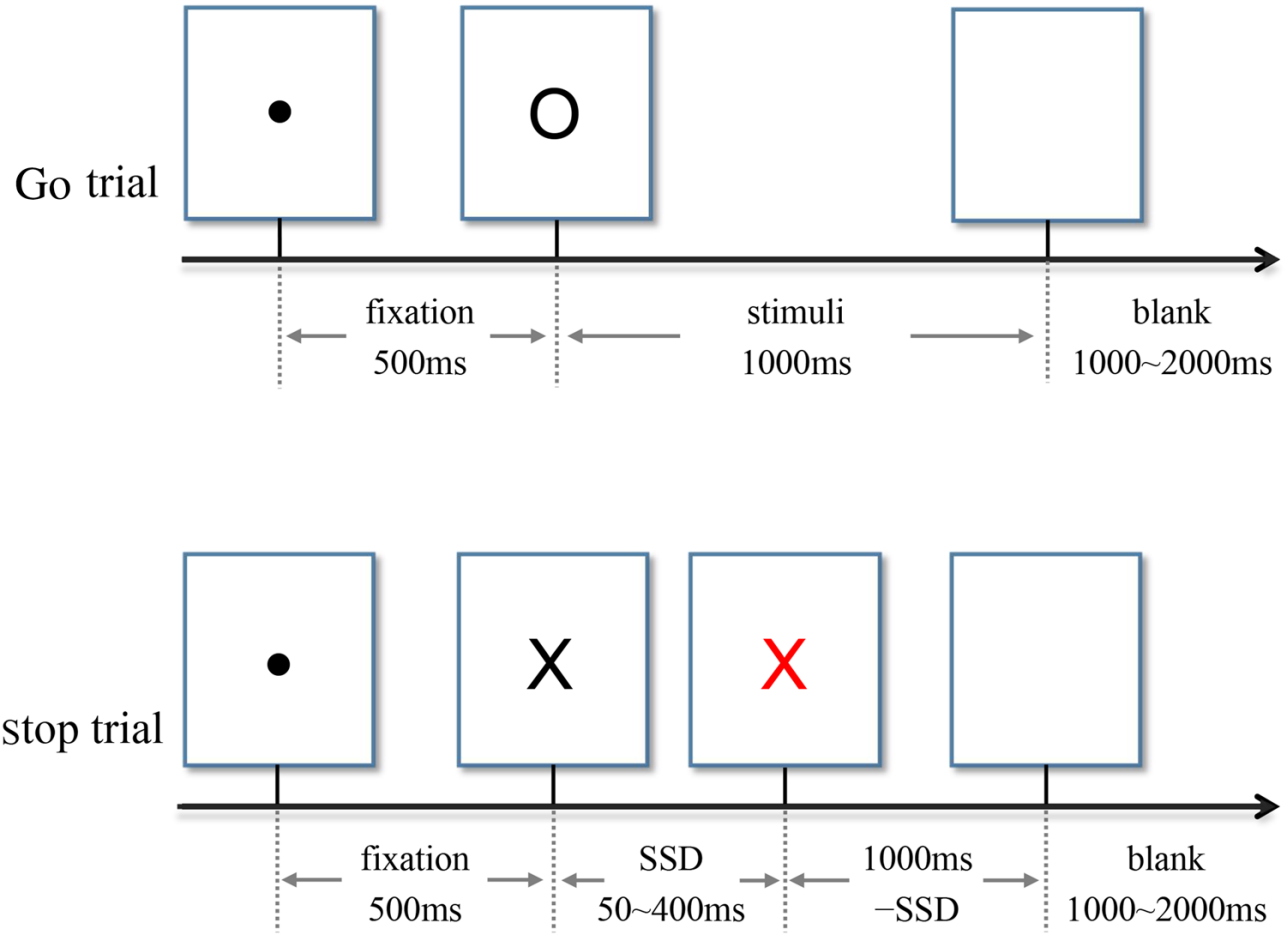
Two types of trials in neutral stop signal task.

Either letter "X" or "O", printed in bold Couiter New, font size 80, was presented at the center of a white background on the computer screen. The participants were asked to press the left key for the letter "X" and the right key for the letter "O". When some letters’ color changed from black to red, however, they were asked to perform no button reaction. This task had 4 blocks, and each block contained 100 trials (30 Stop trails, 70 Go trials). Each trial began with a 500 ms fixation and then a 1000 ms letter "X" or "O". Among them, 30% letters turned red. The time interval from black to red was recorded as the stop signal delay (SSD). The initial SSD was 250 ms. A successful inhibition made the SSD extend 50 ms, while a failed inhibition made the SSD shorten 50 ms. The SSD ranged from 50 to 400 ms. The time interval between trials was set from 1000 to 2000 ms randomly. Stop Signal Reaction time (SSRT) was the Go trials response time minus the signal delay time^66^. The larger the SSRT is, the weaker the impulse inhibition ability is^51^. The task design was in accord with the consensus guide of stop signal task^67^. Before the formal experiment, the participants practiced to familiarize themselves with the experiment rules by exercise trials. The task procedure is showed in Fig. 4.

#### Emotional SST

**Figure 5:**
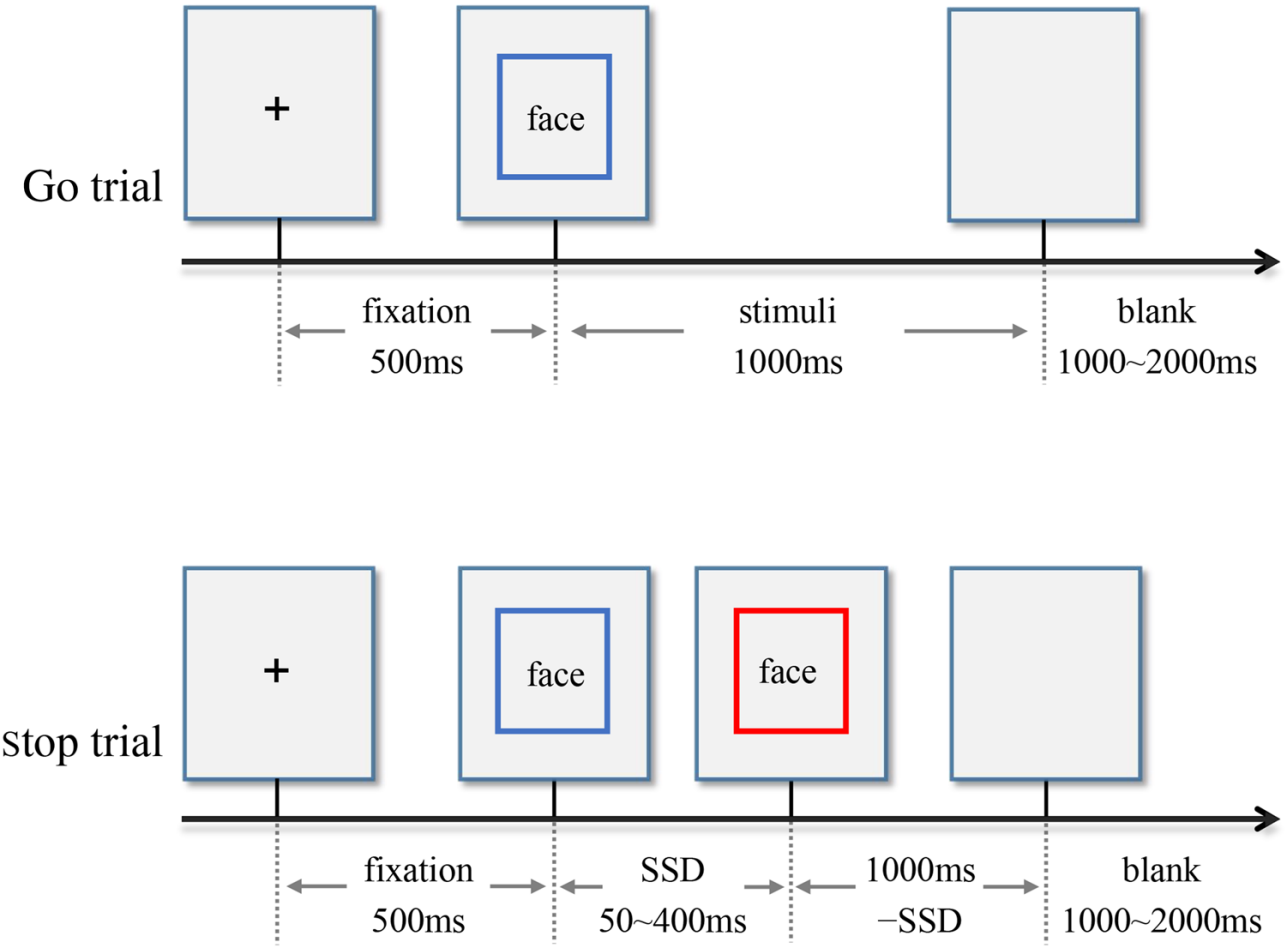
Two types of trials of emotional stop signal task (Face: negative emotion face or neutral emotion face).

In this experiment, 20 negative emotion face images and 20 neutral emotion face images were drawn from the Chinese Affective Picture System^68^. Given that previous studies showed that BPD patients were more sensitive to fearful facial expressions^69^, fearful face images were adopted as the negative emotion stimuli in this study. Images of each emotion type contained 10 males and 10 females. All images were presented on a gray background (visual angle, 6.56° × 8.00°). Subjects were required to judge the gender of the face. If the picture was a female face, participants pressed the left key, otherwise pressed the right key. When some pictures’ border changed from blue to red, however, they were asked to perform no button reaction. The experiment had 5 blocks, and each block contained 80 trials (24 Stop trails, 56 Go trials). Each trial initially displayed a 500 ms fixation, followed by a 1000 ms emotion image. The time during which the pictures’ border changed from blue to red was recorded as the stop signal delay (SSD). The rest of experimental design is the same with neutral SST. The task design is also in accord with the consensus guide of stop signal task^67^. The task procedure is showed in Fig. 5.

### Experimental process

All participants were asked to complete the self-reported scales and then finish the two SST experiments. The order of the two SST experiments was balanced among participants. All experimental tasks were compiled by E-prime 2.0 software, and all stimuli were presented on a 19-inch screen whose resolution was 1024 × 768 pixels. Subjects sat in front of the screen about 60 cm away from the center of the screen, and their eyes were at the same level as the center of the screen.

ERPs data were recorded when participants were performing the SST tasks. The SynAmps amplifier was used with the ESI-64 EEG recording and analysis system and the Ag/AgCl electrode cap manufactured by Neuroscan. Two electrodes on the upper and lower sides of the left eye recorded vertical EOG, and two electrodes on the outside of the two eyes recorded horizontal EOG. The left mastoid M1 electrode was used as the reference electrode. The bandpass of the filter was 0.05–400 Hz. The sampling frequency was 1000 Hz. The impedance at each electrode was kept below 5kΩ. The data was analyzed offline after the continuous recording of EEG.

### Data analysis

Statistical analysis of all data was done in SPSS 17.0.

#### Analysis of psychometric data

The differences in impulsiveness (BIS-11), BPD symptoms severity (BSL-23), depression (CES-D) and anxiety level (STAI) between BPD group and control group were analyzed with independent sample *t* test. Cohen's *d* was used to measure the effect size.

#### Behavioral data analysis of neutral SST

Independent sample *t* test was used to examine the behavioral differences between the two groups. The dependent variables were the correct rate of the Go trials (Go-ACC), the correct response time of the Go trials (Go-RT), the correct rate of Stop trials (Stop-ACC), the stop signal delay (SSD), and the stop signal reaction time (SSRT). Cohen’s d was calculated to indicate effect size.

#### Behavioral data analysis of emotional SST

The repeated-measures variance analysis of 2 (group: BPD group, control group) × 2 (emotion type: negative, neutral) was used to investigate the differences between BPD and control group in emotional impulse inhibition. The dependent variables were the same with neutral SST. The Greenhouse–Geisser method was used to correct the degrees of freedom and *p*-values. The partial η^2^ was used to express the effect size.

#### ERPs data analysis

Pre-analysis: EEG segments with artifacts were removed. Data were filtered with 30HZ low-pass digital filter. The ERPs epoch was defined as from 200 ms prestimulus onset to 800 ms after stimulus onset. Data with amplitude greater than ± 200 μV were excluded. Subjects with less than 30 superpositions were removed. One subject was excluded from the BPD group in the neutral SST, and two subjects were excluded from the control group in the emotional SST.

ERPs data analysis of neutral SST: Two components (N1 and P2) were induced in Go trials, while three components (P1, N2 and P3) were induced in Stop trials. Based on the grand mean waveforms, the peak amplitudes were calculated for each participant in the following time windows: N1 (80–140 ms), P2 (150–250 ms), P1 (120–170 ms), N2 (180–230 ms), P3 (250–400 ms). Data were analyzed by repeated-measures ANOVA of 2 (group: BPD group, control group) × 3 (electrode position: FZ, FCZ, CZ). The dependent variables were the amplitudes and latencies of N1, P2, P1, N2, and P3 components. The Greenhouse–Geisser method was used to correct the degrees of freedom and *p*-values.

ERPs data analysis of emotional SST: Two components (N1 and P2) were induced in Go trials, the time windows of which were N1 (70–120 ms) and P2 (130–200 ms). Five components (P1, N1, P2, N2 and P3) were induced in Stop trials, the time windows of which were P1 (110–160 ms), N1 (170–220 ms), P2 (230–270 ms), N2 (280–330 ms), P3 (350–500 ms). Data were analyzed by repeated-measures ANOVA of 2 (group: BPD group, control group) × 2 (emotion type: negative, neutral) × 3 (electrode position: FZ, FCZ, CZ). The dependent variables were the amplitudes and latencies of P1, N1, P2, N2, and P3 components. Where appropriate, Greenhouse–Geisser method and partial η2 were also used.

## References

[1] APA (2013). Diagnostic and Statistical Manual of Mental Disorders: DSM-5.

[2] Fertuck EA (2006). the impact of anxiety and borderline personality disorder on neuropsychological performance in major depression. J. Pers. Disord..

[3] Winter D (2016). Attention to emotional stimuli in borderline personality disorder—A review of the influence of dissociation, self-reference, and psychotherapeutic interventions. Borderline Personal. Disord. Emot..

[4] Links PS, Heslegrave R, van Reekum R (1999). Impulsivity: Core aspect of borderline personality disorder. J. Pers. Disord..

[5] Jacob G (2010). Impulsivity in borderline personality disorder: Impairment in self-report measures, but not behavioral inhibition. Psychopathology.

[6] Domes G (2006). The influence of emotions on inhibitory functioning in borderline personality disorder. Psychol. Med..

[7] Hurtado MM, Triviño M, Panadero MA, Arnedo M, Tudela P (2018). Comparing adaptation in emotional and non-emotional conflict in patients with schizophrenia and borderline personality disorder. Neuropsychologia.

[8] Barker V (2015). Impulsivity in borderline personality disorder. Psychol. Med..

[9] Paret C, Jennen-Steinmetz C, Schmahl C (2017). Disadvantageous decision-making in borderline personality disorder: Partial support from a meta-analytic review. Neurosci. Biobehav. Rev..

[10] Nigg JT, Silk KR, Stavro G, Miller T (2005). Disinhibition and borderline personality disorder. Dev. Psychopathol..

[11] McCloskey MS (2009). Evaluation of behavioral impulsivity and aggression tasks as endophenotypes for borderline personality disorder. J. Psychiatr. Res..

[12] Albert J (2019). Response inhibition in borderline personality disorder: Neural and behavioral correlates. Biol. Psychol..

[13] van Zutphen L (2020). Impulse control under emotion processing: an fMRI investigation in borderline personality disorder compared to non-patients and cluster-C personality disorder patients. Brain Imaging Behav..

[14] Ruocco AC, Laporte L, Russell J, Guttman H, Paris J (2012). Response inhibition deficits in unaffected first-degree relatives of patients with borderline personality disorder. Neuropsychology.

[15] Aichert DS (2016). Associations between trait impulsivity and prepotent response inhibition. J. Clin. Exp. Neuropsychol..

[16] Unoka Z, Richman M (2015). Neuropsychological deficits in BPD patients and the moderator effects of co-occurring mental disorders: A meta-analysis. Clin. Psychol. Rev..

[17] Chapman AL (2019). Borderline personality disorder and emotion dysregulation. Dev. Psychopathol..

[18] Linehan MM (1993). Skills Training Manual for Treating Borderline Personality Disorder.

[19] Terzi L (2017). Aggressive behavior and self-harm in Borderline Personality Disorder: The role of impulsivity and emotion dysregulation in a sample of outpatients. Psychiat. Res..

[20] Fertuck EA, Dambreville N, Diamond D, Duggal D, Erbe JK (2021). Referential activity differentially mediates expression of positive and negative emotions in borderline personality disorder. J. Psycholinguist. Res..

[21] Stiglmayr CE, Shapiro DA, Stieglitz RD, Limberger MF, Bohus M (2001). Experience of aversive tension and dissociation in female patients with borderline personality disorder: A controlled study. J. Psychiat. Res..

[22] Lynch TR (2006). Heightened sensitivity to facial expressions of emotion in borderline personality disorder. Emotion.

[23] Dyck M (2009). Negative bias in fast emotion discrimination in borderline personality disorder. Psychol. Med..

[24] Vestergaard M (2020). Women with borderline personality disorder show reduced identification of emotional facial expressions and a heightened negativity bias. J. Pers. Disord..

[25] Yen S, Zlotnick C, Costello E (2002). Affect regulation in women with borderline personality disorder traits. J. Nerv. Ment. Dis..

[26] Jacob G (2013). Emotional modulation of motor response inhibition in women with borderline personality disorder: an fMRI study. J. Psychiatry Neurosci..

[27] Ruocco AC (2020). Neurophysiological biomarkers of response inhibition and the familial risk for borderline personality disorder. Prog. Neuropsychopharmacol. Biol. Psychiatry.

[28] Chikazoe J (2010). Localizing performance of go/no-go tasks to prefrontal cortical sub-regions. Curr. Opin. Psychiatry.

[29] Wrege JS (2021). Attentional salience and the neural substrates of response inhibition in borderline personality disorder. Psychol. Med..

[30] Norra C (2003). Enhanced intensity dependence as a marker of low serotonergic neurotransmission in borderline personality disorder. J. Psychiat. Res..

[31] Chen A (2008). The timing of cognitive control in partially incongruent categorization. Hum. Brain Mapp..

[32] Enriquez-Geppert S, Konrad C, Pantev C, Huster RJ (2010). Conflict and inhibition differentially affect the N200/P300 complex in a combined Go/Nogo and stop-signal task. Neuroimage.

[33] Huster RJ, Westerhausen R, Pantev C, Konrad C (2009). The role of the midcingulate cortex as neural generator of the N200 and P300 in a tactile response inhibition task. Hum. Brain Mapp..

[34] Nieuwenhuis S, Yeung N, van den Wildenberg W, Ridderinkhof KR (2003). Electrophysiological correlates of anterior cingulated function in a Go/Nogo task: Effects of response conflict and trial-type frequency. Cogn. Affect. Behav. Neurosci..

[35] Ruchsow M (2008). Response inhibition in borderline personality disorder: event-related potentials in a Go/Nogo task. J. Neural Transm..

[36] Benvenuti SM, Sarlo M, Buodo G, Mento G, Palomba D (2015). Influence of impulsiveness on emotional modulation of response inhibition: An ERP study. Clin. Neurophysiol..

[37] Shen IH, Lee DS, Chen CL (2014). The role of trait impulsivity in response inhibition: Event-related potentials in a stop-signal task. Int. Psychophysiol..

[38] Ramos-Loyo J (2021). Inhibitory control under emotional contexts in women with borderline personality disorder: An electrophysiological study. J. Psychiat. Res..

[39] Vallet W, Neige C, Mouchet-Mages S, Brunelin J, Grondin S (2021). Response-locked component of error monitoring in psychopathy: A systematic review and meta-analysis of error-related negativity/positivity. Neurosci. Biobehav. Rev..

[40] de Bruijn E (2006). Neural correlates of impulsive responding in borderline personality disorder: ERP evidence for reduced action monitoring. J. Psychiat. Res..

[41] Ruchsow M (2006). Electrophysiological correlates of error processing in borderline personality disorder. Biol. Psychol..

[42] Aron AR (2011). From reactive to proactive and selective control: developing a richer model for stopping inappropriate responses. Biol. Psychiatry.

[43] Verbruggen F, Logan GD (2008). Automatic and controlled response inhibition: associative learning in the go/no-go and stop-signal paradigms. J. Exp. Psychol. Gen..

[44] Schachar R (2007). Restraint and cancellation: multiple inhibition deficits in attention deficit hyperactivity disorder. J. Abnorm. Child. Psychol..

[45] Patton JH, Stanford MS, Barratt ES (1995). Factor structure of the Barratt impulsiveness scale. J. Clin. Psychol..

[46] Gremaud-Heitz D (2014). Comorbid atypical depression in borderline personality disorder is common and correlated with anxiety-related psychopathology. Compr. Psychiat..

[47] Luca M, Luca A, Calandra C (2012). Borderline personality disorder and depression: an update. Psychiatr. Q..

[48] Carretié L, Mercado F, Tapia M, Hinojosa JA (2001). Emotion, attention, and the “negativity bias”, studied through event-related potentials. Int. Psychophysiol..

[49] Xin Y, Li H, Yuan JJ (2010). Negative Emotion Interferes with Behavioral Inhibitory Control: An ERP Study. Acta Psychol. Sin..

[50] Yuan JJ (2007). Are we sensitive to valence differences in emotionally negative stimuli? Electrophysiological evidence from an ERP study. Neuropsychologia.

[51] Logan GD, Schachar RJ, Tannock R (1997). Impulsivity and inhibitory control. Psychol. Sci..

[52] LeGris J, Links PS, van Reekum R, Tannock R, Toplak M (2012). Executive function and suicidal risk in women with Borderline Personality Disorder. Psychiat. Res..

[53] Delplanque S, Lavoie ME, Hot P, Silvert L, Sequeira H (2004). Modulation of cognitive processing by emotional valence studied through event-related potentials in humans. Neurosci. Lett..

[54] Gajewski PD, Stoerig P, Falkenstein M (2008). ERP–correlates of response selection in a response conflict paradigm. Brain Res..

[55] Potts GF, Patel SH, Azzam PN (2004). Impact of instructed relevance on the visual ERP. Int. Psychophysiol..

[56] Dimoska A, Johnstone SJ, Barry RJ, Clarke AR (2003). Inhibitory motor control in children with attention-deficit/hyperactivity disorder: event-related potentials in the stop-signal paradigm. Biol. Psychiat..

[57] Lei H (2015). Is impaired response inhibition independent of symptom dimensions in obsessive-compulsive disorder? Evidence from ERPs. Sci. Rep..

[58] Kok A, Ramautar JR, De Ruiter MB, Band GP, Ridderinkhof KR (2004). ERP components associated with successful and unsuccessful stopping in a stop-signal task. Psychophysiology.

[59] Krause-Utz A (2016). Delay discounting and response disinhibition under acute experimental stress in women with borderline personality disorder and adult attention deficit hyperactivity disorder. Psychol. Med..

[60] Bøen E (2015). Different impulsivity profiles in borderline personality disorder and bipolar II disorder. J. Affect. Disorders.

[61] Gong YX (1982). Manual for the Wechsler Adult Intelligence Scale Chinese Revision.

[62] Yang HQ (2007). The Chinese Version of the Barratt Impulsiveness Scale, 11th Version (BIS-11). Chin. J. Clin. Psychol..

[63] Yang H (2018). Psychometric properties of the Chinese version of the brief borderline symptom list in undergraduate students and clinical patients. Front. Psychol..

[64] Wang M (2013). Factor structure of the CES-D and measurement invariance across gender in Mainland Chinese adolescents. J. Clin. Psychol..

[65] Shek DT (1993). The Chinese version of State-Trait Anxiety Inventory: Its relationship to different measures of psychological well-being. J. Clin. Psychol..

[66] Logan GD, Cowan WB (1984). On the ability to inhibit thought and action: A theory of an act of control. Psychol. Rev..

[67] Verbruggen F (2019). A consensus guide to capturing the ability to inhibit actions and impulsive behaviors in the stop-signal task. Elife.

[68] Bai L, Ma H, Huang YX, Luo YJ (2005). The development of native Chinese affective picture system—A pretest in 46 college students. Chin. Ment. Health J..

[69] Mitchell AE, Dickens GL, Picchioni MM (2014). Facial emotion processing in borderline personality disorder: A systematic review and meta-analysis. Neuropsychol. Rev..

